# Serum tryptase, mast cells positive to tryptase and microvascular density evaluation in early breast cancer patients: possible translational significance

**DOI:** 10.1186/1471-2407-14-534

**Published:** 2014-07-24

**Authors:** Ilaria Marech, Michele Ammendola, Rosario Sacco, Gennaro Stefano Capriuolo, Rosa Patruno, Rosangela Rubini, Maria Luposella, Valeria Zuccalà, Eufemia Savino, Cosmo Damiano Gadaleta, Domenico Ribatti, Girolamo Ranieri

**Affiliations:** Interventional Radiology Unit with Integrated Section of Translational Medical Oncology, National Cancer Research Centre IstitutoTumori “Giovanni Paolo II”, Bari, Italy; Department of Medical and Surgery Science, Clinical Surgery Unit, University of Catanzaro “Magna Graecia” Medical School, Catanzaro, Italy; Department of Prevention, Section of Animal Health, ASL BAT, Bari, Italy; Department of Medical and Surgery Science, Cardiovascular Disease Unit, University of Catanzaro “Magna Graecia” Medical School, Catanzaro, Italy; Department of Medical and Surgery Science, Pathology Unit, University of Catanzaro “Magna Graecia” Medical School, Catanzaro, Italy; Department of Experimental Oncology, Clinical Pathology Laboratory National Cancer Research Centre IstitutoTumori “Giovanni Paolo II”, Bari, Italy; Department of Basic Medical Sciences, Neurosciences and Sensory Organs, University of Bari Medical School, Bari, Italy; National Cancer Institute “Giovanni Paolo II”, Bari, Italy

**Keywords:** Angiogenesis, Breast cancer, Surrogate marker, Mast cells, Serum tryptase, Tissue tryptase

## Abstract

**Background:**

Tryptase is a serine protease released from mast cells that plays a role in tumor angiogenesis. In this study we aimed to evaluate serum tryptase levels in 105 female early breast cancer patients before (STLBS) and after (STLAS) radical surgical resection, mast cell density positive to tryptase (MCDPT) and microvascular density (MVD).

**Methods:**

STLBS and STLAS were assessed using the UniCAP Tryptase Fluoroenzyme immunoassay. Tumor sections were immunostained with a primary anti-tryptase antibody and an anti-CD-34 antibody by means of immunohistochemistry.

**Results:**

The mean ± 1 standard deviation STLBS and STLAS was 7.18 ± 2.63 μg/L, and 5.13 ± 2.21 respectively and a significant difference between mean levels was found (p = 0.0001) by student t-test. A strong correlation between STLBS and MVD (r = 0.81, p = 0.0001); STLBS and MCDPT (r = 0.69, p = 0.003); and MCDPT and MVD (r = 0.77; p = 0.0001) was found.

**Conclusions:**

Results demonstrated higher STLBS in breast cancer patients, indicating an involvement of MC tryptase in breast cancer angiogenesis. Therefore, serum tryptase levels may play a role as a novel surrogate angiogenic marker predictive of response to radical surgery in breast cancer patients. In this patients setting, it’s intriguing to hypothesize that tryptase inhibitors might be evaluated in clinical trials.

## Background

Published data suggest that mast cells (MCs) have a dual role in the regulatory function between inflammatory and tumor cells. Interestingly, MCs induce tumor development and progression angiogenesis-mediated by means of the release of various angiogenic molecules such as Vascular Endothelial Growth Factor (VEGF), Fibroblast Growth Factor-2 (FGF-2), tryptase, chymase. On the other hand, MCs may induce apoptosis of malignant cells by means of the release of several cytokines such as interleukin-4 and Tumour Necrosis Factor [1–5].

Several experimental studies have already demonstrated that MCs are involved in tumor macroscopic expansion and development [6–9]. Interestingly, it has been also shown that MCs density (MCD) is strongly related to angiogenesis in animal and human malignancies [3, 9–22]. Among pro-angiogenic factors released from MCs tryptase is one of the most powerful. It has been demonstrated that tryptase induces *in vitro* microvascular endothelial cells proliferation in the matrigel assay and displayed *in vivo* the capillary growth in the chick embryo chorioallantoic membrane [23–25].

Tryptase is an agonist of the proteinase-activated receptor-2 (PAR-2) in vascular endothelial cells and breast cancer cells that in turn stimulates their proliferation [24]. Tryptase also induces angiogenesis by release of stored angiogenic factors bound to the extracellular matrix [26–29]. Very little data have been published regarding the role of tissue MCs density positive to tryptase (MCDPT) in breast cancer (BC) angiogenesis and development [7, 8, 30]. Moreover, no reports are available about the role of serum tryptase in BC angiogenesis and as a circulating predictive surrogate marker.

Therefore, in the present study we aimed to evaluate serum tryptase levels in BC patients before (STLBS) and after (STLAS) radical surgical resection and MCDPT and microvascular density (MVD) in a series of tumor tissue from early BC patients to correlate each to other. The possible role of serum tryptase as predictive surrogate marker of radical surgery has been also evaluated. In this context tryptase inhibitors (gabexate and nafamostat mesylate) might be evaluated in adjuvant clinical trials as a new anti-angiogenic strategy.

## Methods

### Study populations

The clinicopathological features of the patients are summarized in the Table 1. A series of 105 BC patients observed at the Clinical Surgery Unit of the “Magna Graecia” University of Catanzaro were selected. Biopsy specimens were collected from 105 female BC patients who had undergone BC surgery. Patients were selected accordingly the presence of a primary, invasive breast tumor (stage T1-T3), the presence or not of metastases in axillary lymph nodes (stage N0-N2), the absence of distant metastases (M0), the presence of unilateral breast cancer and the absence of previous or concomitant primary cancer. Patients were staged according to the International Union Against Cancer Tumor Node Metastasis (UICC-TNM) classification [31]. They not received neo-adjuvant therapies nor other medications that could interfere with serum tryptase levels. Surgical treatment performed was either a modified radical mastectomy (39 patients in which the tumor had a diameter >3 cm) or a quadrantectomy with axillary lymphadenectomy. No patient was subjected to the investigation of sentinel lymph node. Following surgery, a course of 5–6 weeks of radiation therapy (66 patients) was performed. On the basis of clinicopathological features patients were evaluated to receive adjuvant hormonal therapy or chemotherapy or both. Full ethical approval and signed consent from individual patients were obtained to conduct the study. The full name of ethics institutional committee review board that approved our study is: University Hospital Ethics Committee “Mater Domini”, Germaneto, Catanzaro, Italy.

**Table 1:** **MCDPT and MVD expression, STLBS and STLAS levels as a function of clinicopathological characteristics in a series of 105 breast cancer patients**

***Variable***	***No. of patients***	***No. of tumours with high MCDPT*** * ***(%)***	***No. of tumours with high MVD*** ** ***(%)***	***No. of tumours with high STLBS*** *** ***(%)***	***No. of tumours with high STLAS*** **** ***(%)***
*Age, years*					
Range 26-86	105				
Median 58	105				
< 58 years	48	25 (53)	29 (58)	26 (55)	27 (56)
≥ 58 years	57	33 (58)	29 (51)	31 (54)	27 (48)
*Menopausal status*					
Premenopausal	43	24 (55)	21 (49)	23 (53)	25 (59)
Postmenopausal	62	36 (58)	31 (50)	32 (52)	34 (55)
*Hystological type*					
Ductal	78	41 (52)	37 (47)	38 (49)	40 (51)
Lobular	27	14 (51)	16 (58)	13 (48)	15 (55)
*Tumour size*					
pT_1_	49	26 (54)	25 (55)	27 (55)	24 (49)
pT_2_	36	16 (44)	19 (52)	18 (51)	17 (47)
pT_3_	20	12 (59)	11 (54)	10 (50)	9 (47)
*Nodal status*					
pN_0_	44	19 (43)	21 (48)	20 (46)	23 (52)
pN_1–2_	61	29 (48)	32 (53)	33 (54)	35 (58)
*Cytohistological grade*					
G_1_	37	19 (52)	22 (60)	21 (58)	18 (48)
G_2_	45	20 (44)	23 (51)	21 (47)	22 (49)
G_3_	23	12 (54)	10 (45)	11 (50)	13 (56)
*Estrogen receptor status*					
Negative	29	16 (55)	17 (58)	13 (46)	14 (55)
Positive	76	37 (49)	42 (55)	36 (48)	41 (54)
*Progesteron receptor status*					
Negative	34	19 (55)	16 (47)	18 (53)	17 (50)
Positive	71	36 (51)	35 (49)	39 (55)	37 (52)
*c-erbB-2 status*					
Negative	69	37 (54)	33 (48)	35 (51)	37 (53)
Positive	36	22 (61)	20 (55)	19 (52)	17 (48)

*Median cut-off value: 8 cells per 400 field.

**Median cut-off value: 23 microvessels per 400 field.

***Median cut-off value: 6.4.

****Median cut-off value: 4.8.

### Sample preparation

Blood samples were collected one day before and one day after surgery. Than samples were dispensed into test tubes with serum separator tubes (Becton Dickinson Vacutainer Systems Hemogard, Plymouth, UK) and left for at least 30 min at room temperature. The samples were subsequently centrifuged at 1,500 × g for 15 min at room temperature and the supernatant was recovered. Patient sera were frozen at -80°C. Starting the analytical phase, sera were thawed to room temperature and mixed thoroughly by vortexing at low speed in order to eliminate any particulate matter affecting reproducible results. Serum tryptase levels were measured by fluoro-enzyme-immunoassay (FEIA) using Uni-CAP100 (Pharmacia Diagnostics AB, Uppsala, Sweden).

### Immunohistochemistry

The histological diagnosis was made on haematoxylin-eosin-stained slides and histopathological grading was performed according to the criteria described by Bloom and Richardson, as well, moderately and poorly differentiated state [32]. For the evaluation of MCDPT and MVD a three-layer biotin-avidin-peroxidase system was utilized [33]. Briefly, six-μm-thick serial sections of formalin-fixed and paraffin-embedded biopsy tumour were cut. Then, sections were microwaved at 500 W for 10 min, after which endogenous peroxidase activity was blocked with 3% hydrogen peroxide solution. Adjacent sections were stained with human-specific monoclonal antibodies anti-tryptase (clone AA1; Dako, Glostrup, Denmark) diluted 1:100 for 1 h at room temperature and anti-CD34 (QB-END 10; Bio-Optica Milan, Italy) diluted 1:50 for 1 h at room temperature, respectively. The bound antibody was visualized using a biotinylated secondary antibody, avidin-biotin peroxidase complex and fast red. Nuclear counterstaining was performed with Gill's haematoxylin no. 2 (Polysciences, Warrington, PA, USA). The primary antibody was omitted in negative controls.

### Morphometrical assay

**Figure 1:**
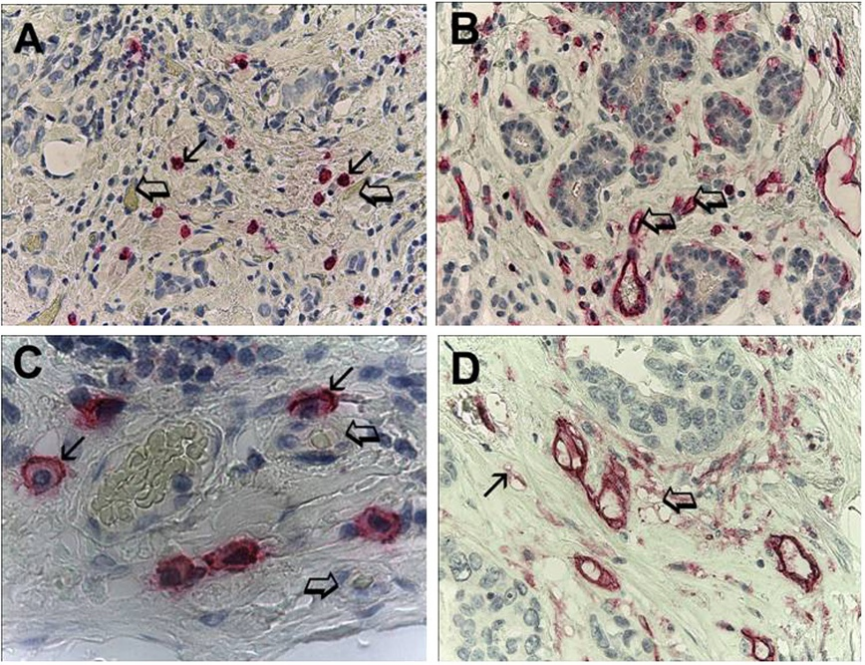
**Immunostaining of breast cancer sections with anti-tryptase and anti-CD34 antibodies. (A)** Small arrows indicate a single red mast cell positive to tryptase. Big arrows indicate a single blood vessel with many red blood cells in their lumen. High MCDPT at x400 magnification (0.19 mm^2^ area). **(B)** Arrows indicate a single red positive microvessel immunostained with a primary anti CD-34 antibody. High MVD at x400 magnification (0.19 mm^2^ area). **(C)** Details of breast cancer section immunostained with a primary anti-tryptase antibody. Small arrows indicate a single red mast cell positive to tryptase, note the blue nucleus of mast cell. Big arrows indicate a single microvessel with a red blood cell in their lumen. X1000 magnification in oil. **(D)** Details of breast cancer section immunostained with a primary anti-CD-34 antibody. Small arrow indicates two red positive microvessels. Big arrow indicates a cluster of microvessels. X1000 magnification in oil.

An image analysis system (Quantimet500 Leica, Wetzlar, Germany) was utilized [33]. The five areas with higher immunostaining (‘hot spots’) were selected at low magnification and individual MCDPT (Figure 1A) and MVD (Figure 1B) were counted at x400 magnification (0.19 mm^2^ area). The details of MCDPT and MVD were evaluated at x1000 magnification in oil (Figure 1C and D, respectively).

### Statistical analysis

MCDPT and MVD mean values ±1 standard deviations (s.d.) were evaluated by two independent observers (V.Z. and G.R.) for each tumor sample and in all series of sections. Correlations between STLBS, MCDPT, and MVD were calculated using Pearson's (r) analysis. The correlations between the above indexes and the clinicopathological features listed in Table 1 were analyzed by the Chi-square test. All statistical analyses were performed with the SPSS statistical software package (SPSS, Inc., Chicago, IL).

## Results

**Figure 2:**
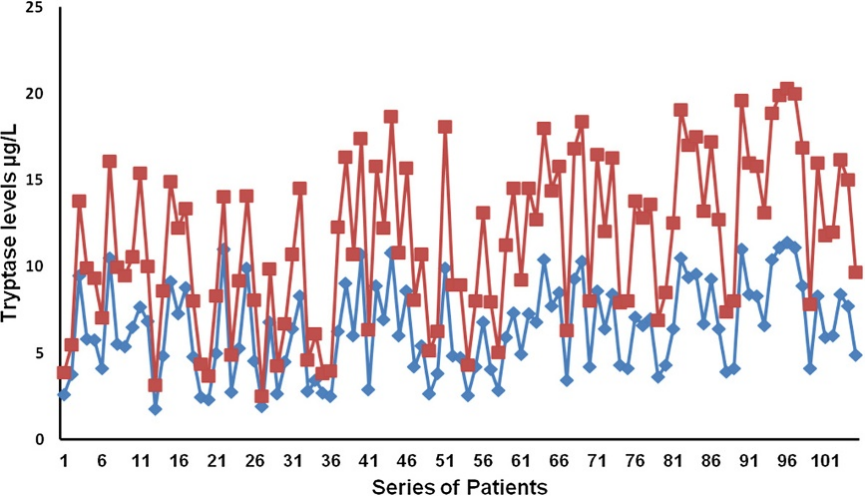
**Tryptase levels before (STLBS) and after (STLAS) surgery in a series of 105 breast cancer patients.**

**Figure 3:**
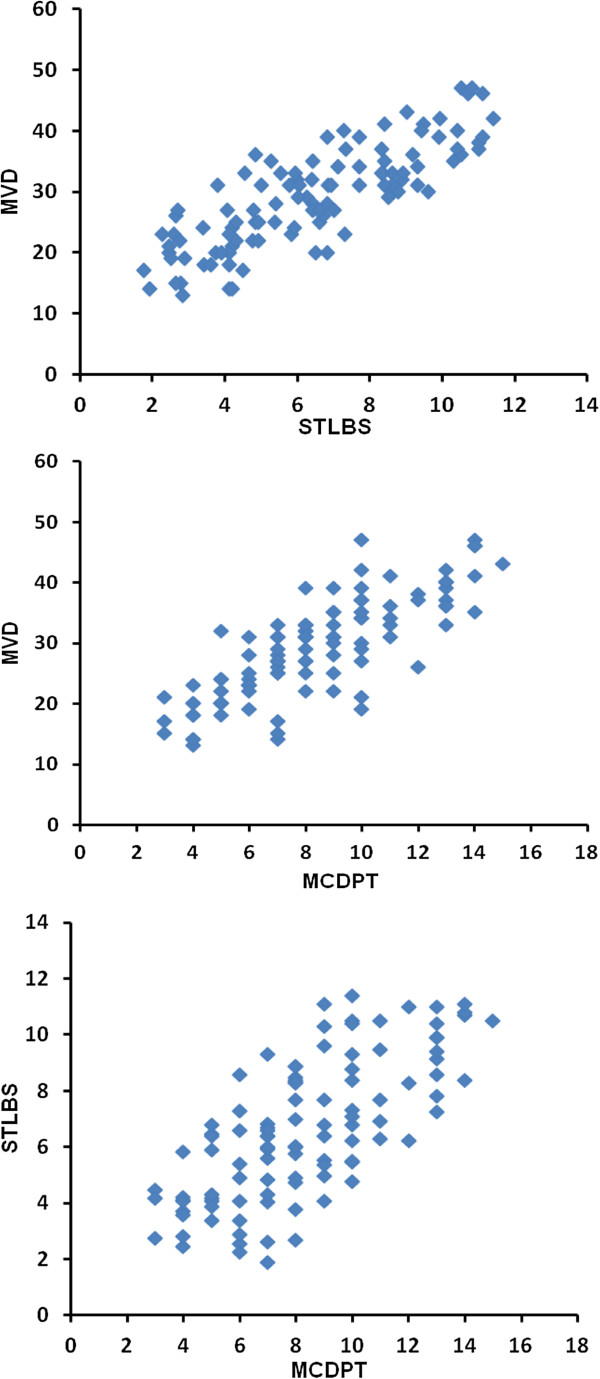
**Correlation analysis between: STLBS and MVD (r = 0.81, p = 0.0001), MCDPT and MVD (r = 0.73; p = 0.001), MCDPT and STLBS (r = 0.60, p = 0.003).**

The mean ± 1 s.d. STLBS and STLAS were 7.18 ± 2.63 μg/L and 5.13 ± 2.21 μg/L respectively, and a significant difference between mean levels was found (p = 0.0001) by t-test (Table 2, Figure 2). The mean ± 1 s.d. of MCDPT and MVD was 8.35 ± 2.99 and 30.11 ± 8.24 respectively (Table 2). A significant correlation between STLBS and MVD (r = 0.81, p = 0.0001), MCDPT and MVD (r = 0.73; p = 0.001), STLBS and MCDPT (r = 0.60, p = 0.003) was found (Figure 3).

**Table 2:** **MCDPT, MVD, STLBS and STLAS means ±1 standard deviations in a series of 105 breast cancer patients**

***MCDPT*** ***400x magnification*** ***(0.19 mm*** ^***2***^ ***area)***	***MVD*** ***400x magnification*** ***(0.19 mm*** ^***2***^ ***area)***	***STLBS μg/L***	***STLAS μg/L***
*8.35 ± 2.99	*30.11 ± 8.24	*7.18 ± 2.63	5.13 ± 2.21

*Mean ± 1 standard deviation.

t-test: p = 0.0001.

Pearson correlation between MVD and STLBS: r = 0.81; p = 0.0001.

Pearson correlation between MVD and MCDPT: r = 0.77; p = 0.0001.

Pearson correlation between STLBS and MCDPT: r = 0.69; p = 0.0003.

## Discussion

It has been well established that MCs are involved in tumor development [6–9] angiogenesis-mediated both in animal and human malignancies [9–22]. However, few data have been published about the role of tissue MCDPT in BC angiogenesis and development [7, 8, 30]. Interestingly, no reports have been published regarding the role of serum tryptase in BC angiogenesis and as a circulating surrogate predictive marker.

In this pilot study we have shown for the first time that STLBS strongly correlates with MCDPT and MVD in primary tumor tissue. Our data also demonstrates that STLAS significantly decrease in BC patients. Due to the release of tryptase from MCs, we suggest that MCDPT in primary BC tumor tissue represents the main source of serum tryptase. In our hypothesis, if primary tumor tissue is completely removed STLAS should decrease in one day due to their approximately 4-h long life-cycle. For these reasons, we detected STLAS 24 h before surgery to evaluate their possible role as a circulating surrogate marker suggesting the presence of tumor tissue, and again 24 h after treatment to confirm its decrease and, as a consequence, its possible expression of the absence of tumor tissue. We elaborate the background of our hypothesis based on previously published pilot data, which suggested an increase of MCDPT in primary tumor tissue. In these studies MCDPT was correlated with MVD, suggesting its role in BC angiogenesis [7, 30]. Our data suggested an involvement of MCs and tryptase in BC angiogenesis. Interestingly, published studies already demonstrated an involvement of tissue MCDPT in other malignancies such as squamous carcinoma, gastrointestinal cancer, non-small cell lung cancer, melanoma, and endometrial carcinoma [9, 11, 12, 18, 19]. However, the above studies did not focus on the changes inSTLs before and after surgery, and no correlation between STLs, MCDPT and MVD was evaluated. It is remarkable that tryptase released from MCs is involved in tumor angiogenesis by several mechanisms. Firstly, tryptase stimulates the formation of vascular tubes in *in vitro* and *in vivo* experimental models; secondly, tryptase is an agonist of the PAR-2 in vascular endothelial cells that, in turn, induces angiogenesis [34]; thirdly, tryptase may stimulate the release of latent angiogenic factors bound to the extracellular matrix [21, 35]. Overall, the above data suggest that tryptase may be a potential surrogate bio-marker of tumor angiogenesis which is able to predict response to surgical treatment.

## Conclusions

If the primary source of tryptase production is no longer existing, after 24 h a significant reduction in STLs should be expected. If elevated STLs persist after surgery, this would suggest that residual tumor tissue remains after surgical resection or, alternatively, that unknown metastases are present. In this context, several tryptase inhibitors, such as gabexate or nafamostat mesilate, may be evaluated in future clinical trials as a new anti-tumor and anti-angiogenic approach.

## Abbreviations

STLBS: Serum tryptase levels before surgery
STLAS: Serum tryptase levels after surgery
MCDPT: Mast cell density positive to tryptase
MVD: Microvascular density
BC: Breast cancer
MCs: Mast cells
VEGF: Vascular endothelial growth factor
FGF-2: Fibroblast growth factor-2
MCD: Mast cells density
PAR-2: Proteinase-activated receptor-2
UICC-TNM: International union against cancer tumor node metastasis
s.d.: Standard deviations.
